# Mothers’ Working Hours and Children’s Obesity: Data from the Korean National Health and Nutrition Examination Survey, 2008–2010

**DOI:** 10.1186/2052-4374-25-28

**Published:** 2013-10-25

**Authors:** Goeun Lee, Hyoung-Ryoul Kim

**Affiliations:** 1 Department of Occupational and Environmental Medicine, Kyung Hee University Hospital, Seoul, Korea; 2 Graduate School of Medicine, The Catholic University of Korea, Seoul, Korea; 3 Department of Occupational and Environmental Medicine, College of Medicine, The Catholic University of Korea, Seoul, Korea

**Keywords:** Obesity, Working hours, Childhood and adolescent obesity

## Abstract

**Objectives:**

The aim of this study is to find the association between mothers’ working hours and obesity of their children according to children’s age and gender.

**Methods:**

This study used data from the second and third year of KNHANES IV and the first year in KNHANES V (2008–2010). We calculate odds ratio (OR) and 95% confidence interval (CI) by using survey logistic regression to assess association of mother’s working hours with overweight or obesity of her children. The model was adjusted with household income, mothers’ education and obesity and mothers’ job characteristics.

**Results:**

13–18 aged boys whose mothers worked under 40 hours per week were higher risk for obesity and overweight (including obesity) than 13–18 aged boys whose mothers worked 40–48 hours. 6–12 aged girls whose mothers worked 49–60 hours per week were more overweight (including obesity) than girls whose mothers worked 40–48 hours per week. 13–18 aged girls whose mothers worked over 60 hours were more overweight (including obesity) than the reference.

**Conclusion:**

This study showed that girls’ obesity was associated with mothers’ long working hours. Long working hours can influence health of workers’ family.

## Introduction

Obesity in childhood and adolescence can lead to obesity in adulthood, and it is well known that obesity causes several diseases, such as hypertension, dyslipidemia, diabetes, cardiovascular disease and colon cancer [1,2]. In particular, this issue emerged as a social problem that exacerbates the burden of future medical problems and costs [3]. One study estimated the social and economic cost of adolescent obesity in Korea was 1,363,800 million Won [4]. Obesity prevalence in aged 2–18 children and adolescent increased from 5.8% in 1995 to 9.7% in 2007 in Korea (6.1% to 11.3%, and 5.5% to 8.0% in boys and girls, respectively) [5].

Childhood and adolescent obesity is affected by home and society which he or she belongs to and genetic factors. One study reported that childhood and adolescent obesity is more related to parents’ socio-economic position (SEP) [6]. As parents’ occupation is an important factor determining the household’s socio-economic position, children’s obesity can be associated with their parent’s job.

With the rapid industrialization for decades in Korea, the number of married women who has occupation is getting higher. Since 2001, the employment rate of married women have been maintained above 50% [7]. In the past, even if a woman before marriage participated in economic activities, she had to quit the job after marriage due to having a baby, housework and parenting at home. But, in recent years, many married women with children are working outside. However, married working women still do a lot of household chores such as taking care of their children, making foods and cleaning. Married woman who has children works both in her workplace and home [8,9]. With regard to the association between mothers’ work and their children’s obesity, many researches were performed in developed countries [10-13]. There is a study that mothers’ working long hours have positively related to child’s obesity [14]. This result explained that working mothers spend less time with their children at home and they do not have enough time to prepare good and slow foods and to guide child’s recreational activities [14,15].

There was no study showing the relevance of the mothers’ work hours and children’s obesity in Korea. In addition, most studies dealing with this issue did not consider children’s age and gender. Therefore, we intended to find out the association between mother’s working hours and children’s obesity according to children’s gender and age, using the data of the Korea National Health and Nutrition Examination Survey (KNHANES).

## Materials and methods

### Subjects

This study used the data of KNHANES in 2008, 2009 and 2010, which are the second and third year of KNHANES IV and the first year in KNHANES V, respectively. The KNHANES is a stratified multistage clustered probability design for selecting representative samples. In total, 29,235 people were involved in this survey. We chose households consisting of “children of a couple” or “children of a single mother”. Among them, by selecting only children and adolescents of the age from 6 to 18 year old and their mothers who have jobs, 2,016 children and 1,220 mothers were selected in this study.

### Methods

All information about household income, children’s age, gender and BMI and mother’s job, working hours per week, work schedule, education and BMI were included in the KNHANES raw data. BMI was calculated by directly measured height and weight. Other information was based on the health questionnaire survey.

The height was measured up to 1 cm, and the weight was measured up to 0.1 kg. BMI was divided the weight (kilogram) by the square of the height (meter) and the second decimal point was permitted. Obesity of children and adolescents abided by the BMI cut-off point considering gender and age based growth curve. Depending on the 2007 Korea Childhood and Youth Standard Growth Chart percentiles, by up to the age of 18, overweight was defined as BMI at 85th percentile or above 85th percentile. Obesity was defined as 95th percentile or above 95th percentile considering gender and age in month. If BMI is 25.0 or over 25, it is also obesity even though he or she was under 95th percentile for age in month [16].

The 2007 Korea Childhood and Youth Standard Growth Chart provided weight, height and BMI according to gender and age in month. We estimated odds ratio in 2 groups, which are comparing obese children (BMI ≥ 95% for gender and age or BMI ≥ 25) with normal weight children (BMI < 95%) and comparing overweight children including obese children (BMI ≥ 85% for gender and age) with normal weight children (BMI < 85% for gender and age). On two groups, “normal” has two different meanings. The first “normal weight” included overweight but the second “normal weight” excluded overweight. For convenience and avoiding confusion, we distinguished “overweight” and “overweight (including obesity)”.

Mothers’ working hours in a week are classified as under 40 hours, 40 to 48 hours, 49 to 60 hours and over 60 hours, respectively.

Household income was categorized into quartiles such as low, middle low, middle high and high. Mother’s education was classified into elementary school, middle school, high school and college or more. Mother’s BMI divided into 2 groups; < 25 was normal and ≥25 was obese. Mother’s occupational classification in KNHANES followed the major groups of the Korean Standard Classification of Occupations (KSCO). We reclassified them into 3 categories, which are white collars (managers, professionals and related workers, office clerks), pink collars (service and sales workers) and blue collars (skilled agricultural, forestry and fishery workers, crafts and related workers, equipments, machine operating and assembling workers, elementary workers and military officers). Status of shift work was classified into day work and shift work (all types of shift work, except day work).

This study received an exemption from screening by the Institutional Review Board of Songeui Medical Campus, The Catholic University of Korea, Seoul, Korea (ID: MC12EASI0158).

### Statistical analysis

KNHANES IV and V data were made of stratified multistage clustered probability design and all statistical analyses incorporated into survey weights. First, we carried out frequency analysis for mothers’ and children’s general characteristics and prevalence. Children and adolescents were stratified by age (6–12 years old and 13–18 years old) and gender. Survey logistic regression was used to calculate odds ratio (OR) and 95% confidence interval (CI) to assess the association of mother’s working hours with overweight or obesity of her children. We adjusted household income, mothers’ education and obesity, mothers’ job characteristics – occupational classification and shift work. All statistical analysis were performed using IBM SPSS STATISTICS version 20.0 and we considered P < 0.05 to be statistically significant.

## Results

The general characteristics of subjects are shown in Tables 1 and 2. Among children, obesity prevalences were 12.8% in boys and 7.7% in girls, respectively (Table 1). In mothers, mothers who work less than 40 hours per week was 38.8% and mothers working 49 hours or more per week were 27.1% (Table 2).

**Table 1:** General characteristic of the children (n = 2,016)

	**Total**	**Male**	**Female**	**P-value**
	**N(%**^**+**^**)**	**N(%**^**+**^**)**	**N(%**^**+**^**)**	
Total	2,016(100.0)	1,058(100.0)	958(100.0)	
Age (years)				0.774
6-12	1,051(44.0)	549(43.6)	502(44.4)	
13-18	965(56.0)	509(56.4)	456(55.6)	
Body Mass Index (kg/m^2^)				0.008
Underweight	121(6.3)	54(5.5)	67(7.4)	
Normal weight	1,534(76.1)	799(75.6)	735(76.8)	
Overweight	167(7.1)	81(6.1)	86(8.1)	
Obese	194(10.4)	124(12.8)	70(7.7)	
Household income				0.477
Low	145(8.2)	79(7.8)	66(8.6)	
Middle low	480(26.1)	236(25.4)	244(27.0)	
Middle high	719(35.7)	367(35.2)	352(36.3)	
High	662(30.0)	371(31.7)	291(28.2)	
Missing	10(0.0)	5(0.0)	5(0.0)	

+ column and estimated percentage.

**Table 2:** General characteristic of the mothers (n = 1,220)

		**Mothers**
		**N(%¶)**
Age (years)	Mean ± S.E.†	41.21(±0.158)
Body Mass Index (kg/m^2^)	< 25	907(75.5)
	≥25	289(24.5)
Education	Elementary school	48(4.5)
	Middle school	103(9.2)
	High school	694(56.7)
	Some college or more	375(29.6)
Job║	Managers	9(0.8)
	Professionals and related workers	272(21.5)
	Office clerks	188(15.0)
	Service workers	190(15.8)
	Sales workers	230(19.5)
	Skilled agricultural, forestry and fishery workers	38(2.4)
	Crafts and related workers	54(4.5)
	Equipments, machine operating and assembling workers	33(2.3)
	Elementary workers	206(18.1)
	Military officer	0(< 0.1)
Working hours per week	< 40	459(38.8)
	40-48	422(33.9)
	49-60	193(15.7)
	>60	145(11.6)
Work schedule	Day work	991(86.5)
	Shift work	156(13.5)

† standard error, ¶ estimated percentage, ║by Korean Standard Classification of Occupations, 6th version.

Mostly, obesity and overweight (including obesity) prevalence in children whose mothers worked 40 to 48 hours was low, while obesity or overweight (including obesity) prevalence was higher in children whose mothers worked over 49 hours or under 40 hours (Table 3).

**Table 3:** Prevalence of children’s obesity and overweight in their mothers’ working hours per week according to age, gender

**Age**	**Gender**	**Mothers’ working hours**	**Total N(%**^**++**^**)**	**Obese N(%**^**++**^**)**	**P-value**	**Overweight and obese N(%**^**++**^**)**	**P-value**
6-12	Male				0.692		0.362
	(N = 548)	< 40	244(100.0)	25(10.0)		47(19.8)	
		40 ~ 48	172(100.0)	12(8.2)		40(23.4)	
		49 ~ 60	76(100.0)	6(11.8)		15(22.9)	
		>60	56(100.0)	4(5.7)		9(11.1)	
	Female				0.078		0.192
	(N = 502)	< 40	222(100.0)	12(5.4)		38(18.3)	
		40 ~ 48	174(100.0)	6(3.8)		25(13.6)	
		49 ~ 60	67(100.0)	9(12.7)		20(26.2)	
		>60	39(100.0)	5(11.2)		8(17.8)	
13-18	Male				0.006*		0.008*
	(N = 509)	< 40	152(100.0)	33(24.0)		39(26.5)	
		40 ~ 48	185(100.0)	19(9.8)		25(12.1)	
		49 ~ 60	94(100.0)	11(9.8)		13(11.8)	
		>60	78(100.0)	14(20.4)		17(22.4)	
	Female				0.588		0.356
	(N = 456)	< 40	145(100.0)	11(11.1)		19(16.7)	
		40 ~ 48	165(100.0)	12(5.9)		18(9.6)	
		49 ~ 60	80(100.0)	8(9.3)		13(15.2)	
		>60	66(100.0)	7(10.6)		15(19.6)	

++ row and estimated percentage.

*p-value < 0.05.

Table 4 shows ORs of children’s obesity and overweight (including obesity) according to mothers’ working hours per week. Among 6–12 year-old aged boys, overweight (including obesity) decreased in children whose mother worked over 60 hours per week. OR was 0.24 (95% CI: 0.06-0.89). 13–18 aged boys whose mothers worked under 40 hours per week were higher risk for obesity and overweight (including obesity) than 13–18 aged boys whose mothers worked 40–48 hours. OR was 4.23 (95% CI: 1.79-10.00) in obesity and 3.83 (95% CI: 1.76-8.31) in overweight (including obesity). 6–12 aged girls whose mothers worked 49–60 hours per week were more overweight (including obesity) than girls whose mothers worked 40–48 hours per week. OR was 2.51 (95% CI: 1.03-6.10). 13–18 aged girls whose mothers worked over 60 hours were more overweight (including obesity) than the reference. OR was 2.62 (95% CI: 1.04-6.62).

**Table 4:** Odds ratios of children’s obesity and overweight in their mothers’ working hours per week stratified by age, gender

**Childrens’**		**Mothers’ working hours**	**Obese odds ratio (95% CI)§**		**Overweight and obese odds ratio (95% CI)§**	
**Age**	**Gender**		**Crude OR**	**Adjusted OR†**	**Crude OR**	**Adjusted OR†**
6-12	Male	< 40	1.25 (0.51-3.05)	1.40 (0.54-3.64)	0.81 (0.48-1.36)	0.73 (0.40-1.33)
		40 ~ 48	1	1	1	1
		49 ~ 60	1.50 (0.42-5.40)	1.59 (0.44-5.74)	0.97 (0.44-2.16)	0.94 (0.39-2.27)
		> 60	0.68 (0.15-2.99)	0.37 (0.06-2.35)	0.41 (0.15-1.10)	0.24 (0.06-0.89)
	Female	< 40	1.42 (0.54-3.70)	1.51 (0.51-4.41)	1.42 (0.77-2.62)	1.14 (0.57-2.30)
		40 ~ 48	1	1	1	1
		49 ~ 60	3.65 (1.09-12.22)	3.40 (0.86-13.54)	2.26 (1.06-4.85)	2.51 (1.03-6.10)
		> 60	3.17 (0.75-13.37)	3.24 (0.63-16.82)	1.38 (0.52-3.67)	1.35 (0.43-4.28)
13-18	Male	< 40	2.89 (1.36-6.15)	4.23 (1.79-10.00)	2.62 (1.31-5.25)	3.83 (1.76-8.31)
		40 ~ 48	1	1	1	1
		49 ~ 60	0.99 (0.40-2.45)	1.15 (0.42-3.11)	0.97 (0.44-2.14)	1.14 (0.48-2.72)
		> 60	2.34 (0.97-5.66)	2.39 (0.90-6.39)	2.10 (0.92-4.78)	2.09 (0.82-5.32)
	Female	< 40	1.97 (0.65-5.94)	2.20 (0.73-6.65)	1.89 (0.80-4.47)	2.02 (0.84-4.89)
		40 ~ 48	1	1	1	1
		49 ~ 60	1.62 (0.57-4.57)	1.22 (0.36-4.22)	1.69 (0.67-4.29)	1.35 (0.47-3.92)
		> 60	1.87 (0.64-5.50)	1.90 (0.65-5.52)	2.30 (0.94-5.65)	2.62 (1.04-6.62)

§ confidence interval (CI).

† adjusted with children’s age, household income and mothers’ education, mothers’ BMI, occupational classification and shift work.

## Discussion

In this study, children’s overweight (including obesity) increased among girls whose mothers worked longer and boys whose mothers worked shorter. Researchers in North America and Europe showed that longer working hours of mothers increased BMI of their children [11,12,17-19]. The reason for such results was analyzed as the following; long working hours of mothers made lacking of time for taking care of their children. So, their children spend more time to watch TV, do less physical activity and eat unbalanced diet [18]. Compared with non-working or part time working mothers, full time working mothers are likely to have their children with overweight and obesity [20].

In our study, we found three interesting results. First, obese and overweight boys whose mothers work under 40 hours per week were more prevalent among 13–18 years old boys. These patterns are shown similarly in other age and gender groups (not significant statistically). Employment rate of Korean married women was very low if they had children under 6 [7,9,17]. Raising small children interrupted mother’s occupations, while mother’s employment rate increased when their children became school-aged or adolescent for their children’s education cost. However, married women whose career was disconnected are usually hired as part-time or non-regular employees. It makes them working under 40 hours in a week [17,21]. It is possible to assume that social economic position of mothers who work under 40 hours can be relatively low in Korea. In Korean studies, married women who worked as part-time or non-regular employment were much more prevalent than unmarried women or married men. Such women were relatively low-educated and service-sales workers. The women in the case of highly educated -college or more- engaged in education service, mostly [9,22,23]. Part-time working married women in service-sales were hired in order to respond to changes in seasonal workload and worked low level of difficulty and simple tasks, while part-time working married women in education service were hired in order to cut down labor costs or substitute regular worker. One study reported their average contract periods were 6.8 months. So, in any cases, part-time working married women were employed as day laborer or temporary workers and their jobs were unstable and paid low wages [23]. Low SEP of mothers can be associated with their children’s obesity. In developed countries, childhood and adolescents of high SEP groups usually consume more vegetables and fruits, which are low calories, than low SEP group [24]. Additionally, children of low income households do eating higher cholesterol intake, binge eating, and more TV watching [25,26]. Also, SEP of household is associated with physical activity of adolescence. High parental education was related with increased physical activity of adolescents and decreased TV watching time [27,28].

The second interesting result was decreasing obesity and overweight of 6–12 year-old boys whose mothers worked over 60 hours, differed from other age and girls. Working over 60 hours a week is very long time. Mothers may go to work very early before their children finish breakfast or return home at very late time after pasting supper time. Young boys’ meals can be neglected under not taking care of mothers for a long time. Adolescents can solve meals without mothers’ help, and young girls can take care of themselves relatively compared to boys of same ages. So, we thought that young boys eat very irregular meals or skip meals. But, more researches about this issue will be needed.

Finally, in 6–12 year-old girls whose mothers work 49 hours or more per week, prevalence of obesity and overweight was much higher. Girls are usually more influenced by their mothers than boys. So, girls whose mothers spend less time with them possibly do less physical activity, and watch TV longer. However, boys are usually outgoing and do more physical activities without mothers’ control, when their mothers work longer. And adolescent girls, compared to adolescent boys, usually do not participated in moderate to high intensity exercise and spend more time watching TV and become less physical activity as grade increases [29,30]. This can be an explanation for this difference according to gender.

The risk for obesity increases in the children and adolescents with using computer and watching TV longer, short sleeping time, and fewer intakes of fruit [31-33]. Working mothers spend less time for cooking, eating with their children, playing, and having leisure time with their children [15,18,34]. It is possible for them to supervise the daily activities of children less [14]. Full-time working mothers who work harder usually may buy instant foods, processed foods, and high calorie food for their families. Furthermore, they rarely serve fruits and vegetables, while they eat high caloric foods [35-37]. In many working mothers, whose working hours are long or who do shift work, it is more frequent that family members tend to eat out and often skip meals and overeat later [38,39]. In the UK, 8–13 year-old children whose mothers are full-time employed spend more time on watching TV than the same aged children whose mothers are non-employed or part-time employed [40].

In this study, we examined the relevance of children’s obesity and mothers’ working hours according to developmental stages - childhood and adolescence - and gender. Through using these stratifications, we intended to find the different effects of mothers’ working hours on their children’s obesity according to gender and age. So, we found the gender difference in this association. This study also used reliable and representative data of Korea. This is strength of our study unlike other studies dealing with this issue.

This study has some limitations. First, we did not consider fathers’ factors such as occupation and BMI. As we know, father’s occupation can represent socioeconomic position of his family. However, information about fathers with growing children in the KNHANES IV and V data is somewhat limited, and especially in single mothers’ households. So, we excluded fathers’ variables - BMI and employment. We considered household incomes instead of fathers’ occupation. Second, we used cross-sectional and secondary data for analysis. We did not consider mothers’ working period. Results may vary depending on child’s developmental stage when the mother has a job. A study showed that mothers’ full-time employment around age 11 affected probability of overweight at age 16 [41]. And, another study reported that the duration of mothers’ employment was associated with higher BMI of their children cumulatively [42]. Additionally, there was no information about mother’s work schedules – whether on weekdays or weekends. There will be a difference in children’s lives depending on whether their mothers work on weekend.

Future studies should consider the working period of mothers, calorie intake, routine diet, physical activity, time of using computer, watching TV, study time of children and adolescents and food selection at home in order to clarify the cause of children’s obesity according to the mothers’ working hours.

## Competing interests

The authors declare that they have no competing interests.

## Authors’ contributions

HRK and GL designed the research. GL collected the data and performed the statistical analysis. HRK and GL interpreted the data and wrote the manuscript. Both authors read and approved the final manuscript.
